# Indications for Medical Cannabis and Their Alignment With US-Wide Disease Prevalences in the US’ Largest Medical Cannabis Program: Cross-Sectional Study

**DOI:** 10.2196/89716

**Published:** 2026-09-08

**Authors:** Sebastian Jugl, Nicole E Smolinski, Tansu Miriam Işılay Aydoğan, Almut G Winterstein

**Affiliations:** 1 Department of Pharmaceutical Outcomes and Policy, College of Pharmacy, University of Florida, 6018 Malachowsky Hall, Gainesville, FL, United States, 1 352-273-6268

**Keywords:** medical marijuana, cannabis, qualifying condition, prevalence, cross-sectional studies, medical marijuana use registry

## Abstract

This research letter compared the prevalence of qualifying conditions among patients certified to use medical cannabis in Florida to population-based prevalence estimates for the same conditions and observed marked discrepancies. These findings highlight conditions that appear to be significant reasons for patients to consider medical cannabis, providing important research priorities for future research on its effectiveness and safety.

## Introduction

The participation in medical cannabis (MC) programs has expanded rapidly in the United States—from 678,408 registered patients in 2016 to 2,974,433 in 2020 and approximately 3,641,949 in December 2024; in Florida alone, 921,698 patients were participating in the statewide MC program as of August 2025 [1-3]. Given the rapid expansion and persistent evidence gaps on therapeutic benefits and risks, rigorous studies of MC safety and effectiveness are urgently needed [4-6]. Understanding patients’ and certifying providers’ preferences can help identify high-priority research areas, for example when qualifying medical conditions (QMCs) with limited evidence on medical cannabis safety and effectiveness are overrepresented among certified patients relative to their prevalence in the general population. While prior studies report the most frequently certified conditions in medical cannabis programs, little is known about how conditions for which MC is used aligns with disease prevalences, and hence patient and provider preferences across different conditions to explore medical cannabis as alternative or supplemental treatment [2,7]. The objective of this study was to compare the prevalence of QMCs among medical cannabis patients in Florida to population-based prevalences of these conditions.

## Methods

We analyzed data from the Florida Medical Marijuana Use Registry to identify QMC among patients with a MC certification in 2025.

The Medical Marijuana Use Registry includes data from all Florida patients who were certified by a physician to receive MC for one or more legally QMCs (the condition that is intended to be treated). Data is generated via standardized online templates from physicians authorized to order medical cannabis for patients. We excluded records with a missing patient identifier or recorded sex, non-resident status or implausible certification dates (closure date preceding the start date).

We extracted US-specific disease prevalences from peer-reviewed literature and scientific reports, focusing on Florida or, if unavailable, the southern United States, or the United States. We prioritized reports that estimated prevalence across the entire population over reports for selected subpopulation, eg, only those with private health insurance.

Allowing multiple QMCs per patient, we calculated condition-specific prevalences by dividing the number of patients with a specific qualifying medical condition on their MC certification in 2025 by the total number of patients with MC certification in 2025. Patients with multiple QMCs were only counted once in the denominator. We then computed prevalence ratios by dividing the Medical Marijuana Use Registry data-derived prevalence for each qualifying medical condition by the population-based prevalence of this condition in Florida.

We supplemented reported prevalence with the level of evidence supporting the effectiveness of medical cannabis for each condition based on the National Academy of Sciences, Engineering, and Medicine report from 2017 that comprehensively investigated the evidence for health effects in cannabis and cannabinoids and an update from a recent published comprehensive mapping review [4,5]. Data analysis was conducted with SAS software (version 9.4).

### Ethical Considerations

This study was approved by the University of Florida Institutional Review Board (IRB202002648) and Florida Department of Health IRB [2020‐101-UFL]. Informed consent was waived. Data in this research did not involve participant compensation. Confidentiality of the data was ensured through anonymity and stringent security measures.

## Results

Of 779,673 qualifying patients in the Medical Marijuana Use Registry in 2025, 8493 (1.09%) were excluded: 7304 (0.94%) nonresidents, 1186 (0.15%) with implausible certification dates, and 3 (<0.01%) with missing recorded sex—yielding 771,180 qualifying patients. The median age was 46.0 years (IQR 34.0‐61.0); 342,258 (44.38%) were female and 428,922 (55.62%) were male. The most prevalent QMCs per 100,000 patients certified to use medical cannabis in Florida in 2025 (Table 1) were posttraumatic stress disorder (PTSD) (81,519), chronic pain (24,081), and multiple sclerosis (12,938). The three QMCs with the lowest prevalence per 100,000 certified patients were acquired immunodeficiency syndrome (AIDS) (174), amyotrophic lateral sclerosis (ALS) (302), and human immunodeficiency virus (HIV) (860). When compared to population-based prevalences, most conditions were more common in the Medical Marijuana Use Registry.

We observed pronounced variations in the ratio of Medical Marijuana Use Registry-based and general population prevalences across QMCs. Notably, ALS demonstrated the highest ratio (77.4), indicating a substantially higher representation among medical cannabis users. This was followed by MS (47.5), and Crohn’s disease (10.4). In contrast, chronic nonmalignant pain (1.0) had similar, and conditions like AIDS (0.6) had lower representation in the Medical Marijuana Use Registry than in the general population. The level of evidence supporting effectiveness of medical cannabis as treatment was substantial for epilepsy, multiple sclerosis, and chronic non-malignant pain, and limited or insufficient for the remainder.

**Table 1.** Qualifying medical condition prevalence in the 2025 Medical Marijuana Use Registry and ratio of Medical Marijuana Use Registry-based prevalence estimates to population-based prevalence estimates of qualifying medical condition, and level of evidence. Cross-sectional descriptive analysis of qualifying medical conditions recorded in the Florida Medical Marijuana Use Registry among Florida residents in 2025. For each qualifying condition, the table reports the estimated US-wide prevalence, the registry-based prevalence among Floridians, the ratio of registry-based to population-based prevalence, and the level of evidence supporting therapeutic effects of medical cannabis for that condition.

Qualifying condition	Estimated US-wide prevalence of the condition (per 100,000)	Medical Marijuana Use Registry based prevalence of Floridians with the condition in 2025 (per 100,000)	Ratio of Medical Marijuana Use Registry based prevalence to population-based prevalences	Level of evidence of therapeutic effect
Cancer	591^a^	5917	10.0	None or insufficient^b^
Epilepsy	1700^c^	1441	0.9	Substantial^d^
Glaucoma	1620^e^	1301	0.8	Limited evidence
HIV^f^	704^g^	860	1.2	Limited evidence
AIDS^h^	308^g^	174	0.6	Limited evidence
PTSD^i^	4700^j^	81,189	17.3	Limited evidence
ALS^k^	3.9^l^	302	77.4	None or insufficient
Crohn’s disease	258^m^	2683	10.4	None or insufficient
Parkinson disease	412^n^	579	1.4	None or insufficient
Multiple sclerosis	273^o^	12,938	47.5	Substantial^p^
Chronic nonmalignant pain	24,300^q^	24,081	1.0	Substantial

a International Agency for Research on Cancer. Cancer Today: GLOBOCAN 2022, version 1.1. Global Cancer Observatory. February 8, 2024 [8]. [May 13, 2026].

b Based on effectiveness for disease modification.

c Florida Department of Health. Behavioral Risk Factor Surveillance System (BRFSS) data viewer. FLHealthCHARTS [9].

d Evidence primarily based on Dravet and Lennox-Gastaut syndrome.

e Ehrlich JR et al. [10].

f HIV: human immunodeficiency virus.

g Centers for Disease Control and Prevention [11].

h AIDS: acquired immunodeficiency syndrome.

i PTSD: post-traumatic stress disorder.

j Goldstein RB et al [12]

k ALS: amyotrophic lateral sclerosis.

l Mehta P et al [13].

m Lewis JD et al [14].

n The Lewin Group, Inc. [15]

o Wallin MT, et al [16].

p Evidence based on patient-reported spasticity outcomes.

q Lucas JW, Sohi I [17].

## Discussion

In this cross-sectional study, we found that the prevalence of QMCs among certified MC users in Florida differed from general population disease prevalences suggesting that patients’ and physicians’ perceptions of MC’s role as alternative treatment differs across conditions [18]. Indications with limited or substantial evidence for medical cannabis’s effectiveness were evenly distributed among under- and overrepresented conditions, indicating that the level of evidence for medical cannabis’s effectiveness is not the dominant factor in driving certifications for MC. This finding aligns with previous research suggesting that the evidence base for a qualifying condition appears not to be the main aspect facilitating certifications for medical cannabis [7].

Our study’s limitations include its geographic confinement to Florida, inability to ascertain patients’ primary reason for MC use as this might be different from certifiable conditions under Florida law, and variation in physician certification practices of registry recorded QMCs. We also relied on prevalence estimates from different sources that were not always specific to Florida and used different approaches. Future studies should test reproducibility in other states, develop state-specific general-population prevalence estimates for QMCs to improve comparisons, and examine the relationship between patients’ QMCs in the Medical Marijuana Use Registry and patients’ documented medical histories.

To conclude, our findings define important research priorities regarding the evaluation of both effectiveness and safety of MC for conditions that appear to be important reasons for patients to consider MC. Future studies to investigate the effectiveness and safety of cannabis should consider these research priorities.

## Abbreviations

AIDS: acquired immunodeficiency syndrome
ALS: amyotrophic lateral sclerosis
HIV: human immunodeficiency virus
MC: medical cannabis
PTSD: posttraumatic stress disorder
QMC: qualifying medical condition
